# Study of the Reactivity of Lignin Model Compounds to Fluorobenzylation Using ^13^C and ^19^F NMR: Application to Lignin Phenolic Hydroxyl Group Quantification by ^19^F NMR

**DOI:** 10.3390/molecules25143211

**Published:** 2020-07-14

**Authors:** Esakkiammal Sudha Esakkimuthu, Nathalie Marlin, Marie-Christine Brochier-Salon, Gérard Mortha

**Affiliations:** Univ. Grenoble Alpes, CNRS, Grenoble INP, Institute of Engineering Univ. Grenoble Alpes, LGP2, F-38000 Grenoble, France; nathalie.marlin@grenoble-inp.fr (N.M.); marie-christine.brochier-salon@lgp2.grenoble-inp.fr (M.-C.B.-S.); gerard.mortha@grenoble-inp.fr (G.M.)

**Keywords:** lignin model compounds, organosolv lignin, ^13^C-NMR, ^19^F-NMR, fluorobenzylation, etherification, hydroxyl group quantification

## Abstract

Lignin is an aromatic biopolymer derived from lignocellulosic biomass. Providing a comprehensive structural analysis of lignin is the primary motivation for the quantification of various functional groups, with a view to valorizing lignin in a wide range of applications. This study investigated the lignin fluorobenzylation reaction and performed a subsequent ^19^F-NMR analysis to quantify hydroxyl groups, based on a work developed two decades ago by Barrelle et al. The objectives were to check the assignments proposed in this previous study and to examine the reactivity of various types of lignin hydroxyls with the derivatization agent. Selected lignin model compounds containing phenolic and aliphatic hydroxyls were subjected to the fluorobenzylation reaction, and the obtained reaction medium was analyzed by ^13^C and ^19^F NMR spectroscopy. The model compound results showed that phenolic hydroxyls were totally derivatized, whereas aliphatic hydroxyls underwent minimal conversion. They also confirmed that ^19^F NMR chemical shifts from −115 ppm to −117.3 ppm corresponded to phenolic groups. Then, a ^19^F NMR analysis was successfully applied to Organosolv commercial lignin after fluorobenzylation in order to quantify its phenolic group content; the values were found to be in the range of the reported values using other analytical techniques after lignin acetylation.

## 1. Introduction 

Lignin is an aromatic biomacromolecule which is highly branched and widely available, with a large variety of functional groups such as hydroxyl, methoxyl, carbonyl and carboxyl [1]. In particular, the quantification of the hydroxyl group (aliphatic and phenolic) content in the lignin molecule is of primary importance in analyses of reactivity, solubility and stability [2], which are relevant to applications such as additives, coatings, adhesives and polymer materials [3,4]. However, some of the obstacles to the use of lignins as polymeric materials are heating deformation and phase separation. These issues were successfully overcome by chemical modification of the hydroxyl groups, mainly via esterification and etherification [5,6,7,8]. 

Classically, phenolic functional groups can be quantified after lignin acetylation followed by aminolysis (de-acetylation) [9] using gas chromatography. However, this method has some limitations, e.g., incomplete and time-consuming reaction, and interference by aliphatic hydroxyls in lignin and sugar impurities which lead to a deacetylation process; hence, the true quantification of free phenolic groups is questionable [10]. At present, modern NMR spectroscopic techniques [11,12,13,14] such as ^13^C, ^19^F, ^31^P, and 2D ^1^H-^13^C Heteronuclear Single-Quantum Coherence (HSQC) are the most popular methods in the literature [15,16,17] for the quantification of lignin functionalities. However, direct phenolic group quantification using ^13^C-NMR is relatively hard due to broad C-OH ^13^C chemical shifts and their overlap with other lignin signals. In such cases, acetylation derivatization is mainly performed for the quantification of hydroxyl groups. Moreover, due to the low sensitivity of the ^13^C nucleus, long acquisition times (i.e., more than 12 h), high amounts of derivatized lignin (minimum of 300 mg) and advanced expertise are required for quantitative analysis [18,19]. On the other hand, ^31^P-NMR spectroscopic analysis [16,20,21,22,23,24,25], developed by Argyropoulos and collaborators to quantify phenolic, aliphatic hydroxyl groups and carboxyl groups of lignin in a single analysis, has become a preferred method. Several phosphitylating agents have been used such as 2-chloro-1,3,2-dioxaphospholane [20,21] or 2-chloro-4,4,5,5-tetramethyl-1,3,2-dioxaphospholane (TMDP), which give better signal separation [26], especially between syringyl phenolic hydroxyls and aliphatic hydroxyl groups. The latter reagent also offers reduced reactivity, and thus, enhances the stability of phosphitylated compounds and lignin. Argyropoulos et al. studied the chromophores in mechanical pulps using trimethyl phosphite derivatives with ^31^P-NMR [23,24]. Compared to acetylation, the phosphitylation reaction is faster, since the reagent is introduced directly in the NMR tube containing the lignin to be analyzed, thereby providing a clear description of syringyl and guaiacyl units. However, in the case of using 2-chloro-4,4,5,5-tetramethyl-1,3,2-dioxaphospholane, no distinction can be made between primary and secondary aliphatic hydroxyl groups [26], and the hydroxyl group content tends to be slightly underestimated compared to the data obtained by other analytical techniques [25]. Furthermore, phosphorylated lignin derivatives exhibit poor stability for a longer period of time, and thus require instant acquisition for ^31^P-NMR [27]. 

**Figure 1:**
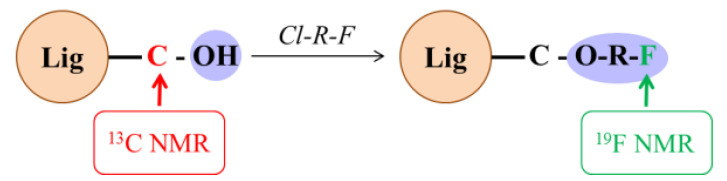
A schematic reaction of lignin fluorobenzylation (*Cl-R-F*—4-Fluorobenzylchloride, Lig—Lignin).

Two decades ago, Barrelle et al. [28,29,30] developed ^19^F-NMR spectroscopic analysis after lignin derivatization using fluorinated compounds to quantify phenolic and aliphatic hydroxyl groups, as well as carbonyl groups (Figure 1). Fluorobenzylation and fluorobenzoylation methods were applied to quantify phenolic hydroxyl groups and aliphatic hydroxyl groups, whereas trifluoromethylphenylhydrazine derivatization enabled carbonyl groups determination [31,32,33]. This particular study did not consider the influence of impurities, for instance, the sugar present in the lignin. Furthermore, the analyses were performed in the early 1990s, and modern ^19^F-NMR instruments can be different; therefore, the signal assignments need to be validated. ^19^F-NMR has some advantages: the derivative is stable, so that it can be reused for other analyses; the natural abundance of 100% and the high sensitivity of the ^19^F nucleus compared to ^13^C-NMR; the ^19^F-NMR acquisition time is quite low (less than 30 min for a lignin sample); and quantitative analyses require only a small amount of lignin derivative (15 mg). In contrast, ^13^C-NMR and ^31^P-NMR require about 100–300 mg and 30 mg, respectively.

**Figure 2:**
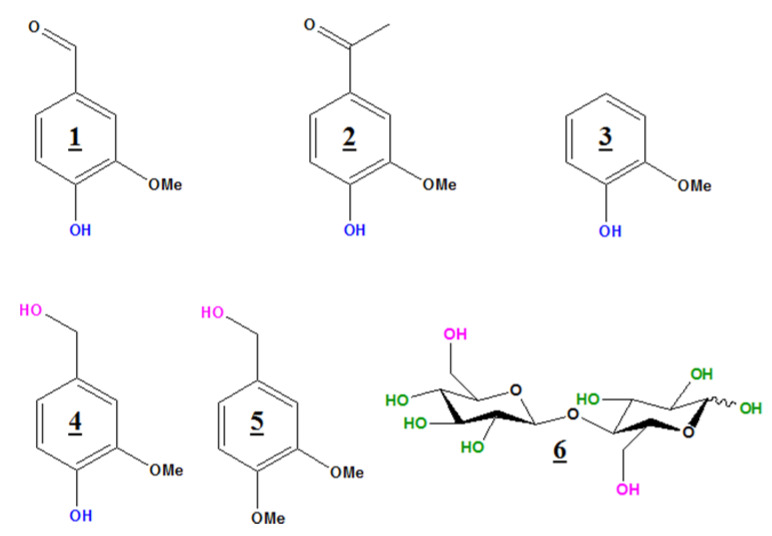
Studied model compounds: Vanillin (**1**), Acetovanillone (**2**), Guaiacol (**3**), Vanillyl alcohol (**4**), Veratryl alcohol (**5**) and D(+) Cellobiose (**6**).

**Figure 3:**
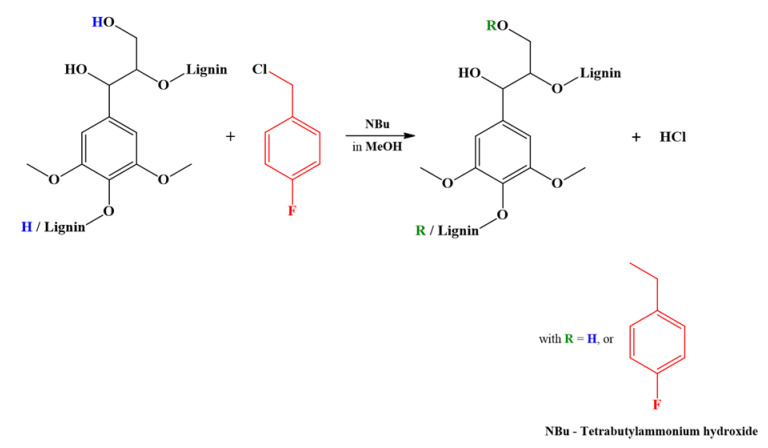
Reaction of lignin fluorobenzylation.

The general ^19^F spectral width is very large (more than 300 ppm). In previous years, since it was easier to detect small spectral widths (especially due to the frequencies filters), NMR instrument technology made it possible to work on spectral foldings, leading to incorrect chemical shift assignments, and sometimes, to reverse shaped signals. Considering recent NMR technological developments, that can now examine the whole spectral window, the present study proposes to re-investigate ^19^F-NMR analysis in the context of a lignin characterization. First, the ^19^F-NMR chemical shift assignments given by Barrelle were verified using various lignin model compounds (phenolic and nonphenolic ones; see Figure 2) after fluorobenzylation (Figure 3). At the same time, the fluorobenzylation reactivities of different hydroxyl were studied using a combination of ^13^C and ^19^F-NMR analyses performed on lignin model compounds. Finally, the hydroxyl groups of a commercial lignin, i.e., Organosolv (ORG) lignin, were quantified using ^19^F-NMR spectroscopy.

## 2. Results and Discussion 

Five lignin model compounds (Figure 2) were selected based on the nature of the hydroxyl groups which can be found in lignins. Vanillin (**1**) and acetovanillone (**2**) contain a phenolic hydroxyl group with a carbonyl function in the α position (aldehyde and ketone respectively). Guaiacol (3) is one of the basic phenolic units of lignin. Vanillyl alcohol (**4**) consists of both aliphatic and aromatic hydroxyl groups, while veratryl alcohol (**5**) contains only one aliphatic hydroxyl group. A carbohydrate model, i.e., D(+) Cellobiose (**6**), was also studied, because commercial lignins are usually contaminated with sugars containing hydroxyl groups (primary and secondary OH) which may also undergo derivatization. In such cases, lignin ^19^F-NMR spectra will contain signals belonging to sugar carbohydrate contaminants; this eventuality will also be examined.

### NMR Analysis

**Figure 4:**
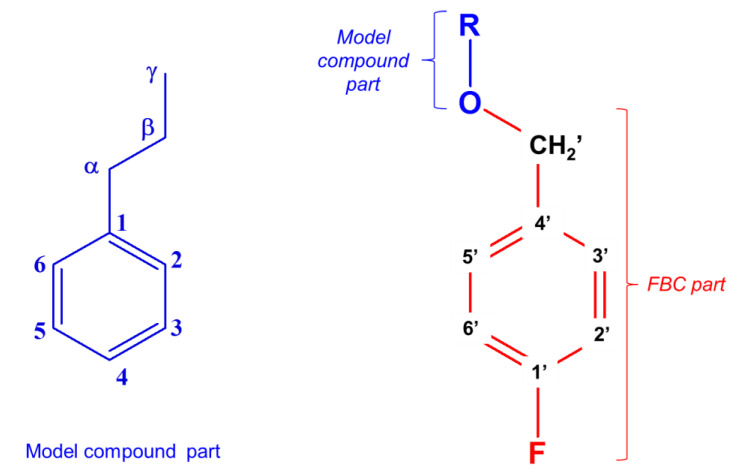
Structure and number assignment of fluorobenzylated model compounds and lignin.

First, the derivatization reagent FBC and all the fluorobenzylated model compounds and their reaction products were quantitatively analyzed by both ^19^F and ^13^C-NMR. The fluorobenzylation reaction is shown in Figure 3. To study this reaction, the following methodology was adopted: The freeze-dried reaction products, recovered from the organic phase without any purification, were first analyzed by ^19^F-NMR. After ^19^F signal detection, ^13^C-NMR analysis was used to confirm, or not, that the F nucleus belongs to the lignin model derivative. This made it possible to elucidate the structure of the derivatized compounds and detect all possible nonfluoro substituted products. The chemical shifts and the specific hyperfine structures due to ^n^J_CF_ couplings were used to determine the presence or absence of a F-**Φ** (para-fluoro phenyl ring originating from FBC) structure. The DEPT sequence indicates the unambiguous assignment of the **C**H_2_′ carbons (Figure 4), and examination of their specific chemical shifts makes it possible to ascertain whether fluoro derivatization has occurred. Chemical shift assignments gave the following results (Table 1). The methylene group of FBC (**C**H_2_Cl) and that of N-Bu (**C**H_2_N) were detected at 45.3 ppm and 57.54 ppm, respectively, in relation to the nature of the heteroatom (Cl or N). The FBC reagent reacts with water (from moisture) and/or methanol (present in N-Bu reagent) to form FBOH and/or FBOMe. Their corresponding signals were seen in the range of 62–63 ppm and 72.81 ppm respectively. Finally, the corresponding fluoroderivatized model compound CH_2_OR signals were in the range of 69–71 ppm.

The reactivity of the fluorobenzylation reaction on lignin model compounds was studied. However, the obtained reaction products were not purified prior to the ^13^C-NMR analysis. The reaction medium contained a mixture of products such as the unreacted starting model compounds, fluorobenzylated model compounds (F-Compound), remaining pure reagent (FBC) and the byproducts FBOH (produced during the reaction of FBC with H_2_O) and FBOMe (produced during the reaction of FBC with MeOH). To quantify each component after fluorobenzylation in the reaction medium, the total composition inside the NMR tube was considered to be 100%. Then, a quantitative ^13^C-NMR analysis was performed on the reaction medium which remained perfectly homogeneous throughout the analysis. In this way, the ratio of each moiety formed during the reaction could be determined (Table 2). The fluorobenzylation reagent FBC purity was controlled by taking a blank ^13^C-NMR spectrum. According to the ^13^C results, FBC contained 94.1% pure FBC and 5.9% FBOH. Similarly, a “FBC blank reaction” was carried out with all the reagents except the model compound. After the reaction, the spectrum indicated the following reaction mixture composition: 7.9% FBOH and 92.1% NBu reagent. No starting FBC signal was detected (see Table 2); this clearly shows that the FBC reagent was totally converted into FBOH. 

**Figure 5:**
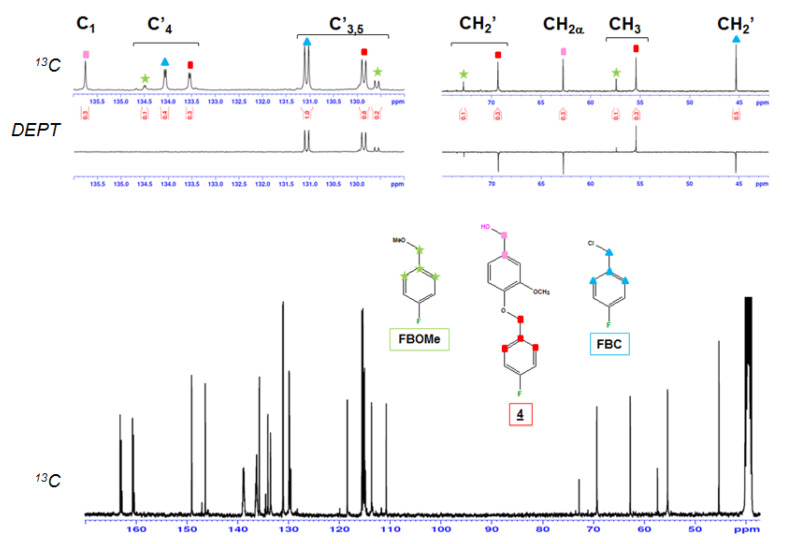
^13^C-NMR spectra of the mixture issued from the fluorobenzylation of vanillyl alcohol (**4**) in DMSO-d6.

For lignin and carbohydrate model compounds, the fluorobenzylation conversion was calculated as follows: Conversion = (F-Compound/(Unreacted pristine compound + F-Compound)) × 100. As seen in Table 2, it is interesting to note that the conversion achieved 100% with the lignin model compounds (**1**), (**2**) and (**3**), as they contain only phenolic hydroxyl groups. In the case of Vanillyl alcohol (**4**), both phenolic and aliphatic hydroxyl groups were present. The absence of a starting model compound shows that its conversion was total (Table 2). Three different compounds were found in the reaction mixture (Figure 5): FBC, with its methylene type carbon at 45.3 ppm, was the most predominant product (53.9%); FBOMe, formed from the reaction of methanol with FBC and its corresponding methyl and methylene group carbon at 57.40 ppm and 72.81 ppm, respectively, represented the minor part (10%); and finally, the remaining approximately 34% was fluoroderivatized vanillyl alcohol. From these results, it may be concluded that the fluorobenzylation conversion of lignin phenolic hydroxyl groups was nearly quantitative, as already seen with previous lignin model compounds (**1**, **2** and **3**). However, the remaining signal at 62.76 ppm corresponding to the methylene carbon covalently bonded to the free aliphatic hydroxyl –CH_2_OH group, and the absence of a signal at 71 ppm corresponding to the –CH_2_OR aliphatic fluoroderivatized group, show that the conversion was due solely to the phenolic hydroxyl (Table 3), with no reaction of the aliphatic hydroxyl group. This was also confirmed by the integral measurements. The integrals of carbons belonging to the model compound part displayed the following values: 0.34, 0.31 and 0.29, corresponding to –OCH_3_ (55.39 ppm), C_1_ (135.75 ppm) and CH_2α_ (62.76 ppm), respectively. Carbons belonging to the FBC part displayed the following values: 0.32, 0.32 and 0.77 corresponding to CH_2_′ (69.33 ppm), C′_4_ (133.56 ppm) and C′_3_ and C′_5_ (129.87 ppm), respectively. This confirmed unambiguously that there was only one FBC moiety per model compound. So, the fluorobenzylation took place on the free phenolic group only, without any reaction on the primary aliphatic hydroxyl function.

**Figure 6:**
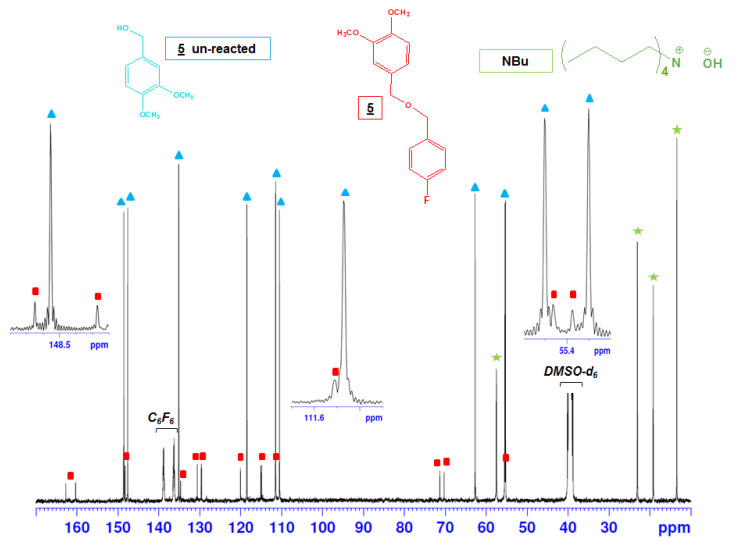
^13^C-NMR spectra of the mixture resulting from the fluorobenzylation of veratryl alcohol (**5**) in DMSO-d6.

For veratryl alcohol (**5**), bearing only one aliphatic hydroxyl and no phenolic one, three products were found (Table 2, Figure 6 and Table 3): unreacted starting veratryl alcohol, with–CH_2_OH and C_1_ signals respectively at 62.74 ppm and at 135.13 ppm, was recovered in the largest quantity (71.5%); Next, 21.4% butyl ammonium was found with its characteristic signals at 13.46, 19.22, 23.09 and 57.54 ppm; This last signal, very close to the chemical shift of the FBOMe methyl group (57.39 ppm), displayed a CH_2_ multiplicity (as given with the DEPT experiment in Figure 6). Nevertheless, CH_3_ multiplicity was expected in the case of FBOMe, confirming the assignment of this signal to the butyl ammonium moiety. Finally, fluoroderivatized veratryl alcohol was recovered as a minor compound (7.1%). The existence of compound (**5**) was confirmed with the characteristic signal of the FBC part, and the chemical shifts modifications of –CH_2_-OR at 71.36 ppm and of C_1_ at 130.61 ppm. Thus, the fluorobenzylation conversion of the veratryl alcohol (**5**), containing only one aliphatic hydroxyl group, was found to be low, i.e., about 9%, and most of the starting model compound remained unchanged. The typical results again suggest that aliphatic hydroxyls hardly react with FBC. Moreover, no more FBC remained after the reaction. Trials were conducted for veratryl compound (**5**) with an increasing FBC stoichiometric ratio and a longer reaction time, i.e., up to 3 days, to further study the reactivity of aliphatic hydroxyls towards fluorobenzylation. It is clearly seen in Table 2 that new conditions did not affect the fluorobenzylation conversion, that remained below 10%. In addition, a high quantity of unreacted FBC was observed, and fluorobenzylation took place with a methanol solvent, resulting in the formation of FBOMe. Therefore, it is evident that the fluorobenzylation was more efficient with phenolic hydroxyl than with primary aliphatic hydroxyl groups.

Similarly, in the case of cellobiose (**6**), the composition of both the aqueous and organic parts recovered after the fluorobenzylation reaction revealed that no signal was obtained for the fluorinated product, nor for the starting carbohydrate model compound (in Table 2). Only butyl ammonium (26%) and FBOH (74%) were detected. The aqueous part results showed that the reaction mixture contained only the starting cellobiose (10%) and NBu reagent (90%). Considering that the total composition inside the NMR tube was 100%, the starting cellobiose (**6**) remained unchanged after the reaction. This clearly indicates that hydroxyls of carbohydrates cannot be fluorobenzylated. Therefore, lignin contamination with sugar will not interfere with the ^19^F-NMR results for lignin derivatization.

**Figure 7:**
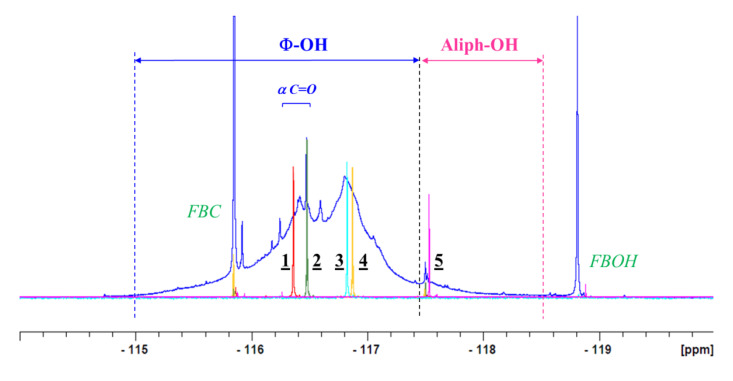
Comparison of ^19^F-NMR spectra of fluorobenzylated ORG lignin (in blue) and derivatized model compounds. (Note: Only phenol derivatization for **4**).

The derivatized model compounds were also analyzed by ^19^F-NMR (same NMR tube as for ^13^C-NMR). According to ^19^F-NMR experiments, only fluorinated compounds can be detected, and hence, no signal remained for the either starting material or for NBu. The reaction mixture compositions (expressed in %) showed some differences between the ^13^C and ^19^F-NMR data. From the ^19^F-NMR (Table 4 and Table 5 and Figure 7), it is clear that all the phenolic hydroxyl groups of compounds (**1**), (**2**) and (**3**) were quantitatively fluorobenzylated, showing signals at −116.36 ppm, −116.49 ppm and −116.89 ppm respectively. Concerning the model compound (**4**), containing both aliphatic and phenolic OH, ^19^F-NMR confirmed that the conversion took place only in the phenolic region (−116.99 ppm), since no peak was detected in the aliphatic region. The conversion of 98% comes from the derivatization of the phenolic hydroxyl group alone. The aqueous part was also verified, but no ^19^F signal was detected, confirming that only phenols could be fluorobenzylated under the experimental conditions. For model compound (**5**), containing only one aliphatic hydroxyl group, after a reaction time of one day, the conversion was very low, i.e., around 16% (Table 4). The resulting signal of the fluoroderivatized compound was detected at −117.50 ppm (Table 5). Even after a longer reaction time (Table 4), F-compound conversion decreased, and simultaneously, the byproduct quantity increased, confirming the ^13^C-NMR results. In the case of the cellobiose (**6**) (Table 4), no ^19^F signal was detected in either the aqueous or organic parts. Moreover, it can be seen that the organic part contained 100% of the FBOH originating from the reaction between FBC and water. This result is consistent with the ^13^C-NMR data. 

After studying the fluorobenzylation conversion of lignin hydroxyl groups, the ^19^F-NMR chemical shift assignments given by Barelle [28,29,30] were tested, since working on folding spectra may have induced incorrect assignments. In the present study, the entire spectral window was examined. The ^19^F-NMR chemical shifts of all investigated fluoroderivatized model compounds are given in Table 5, and the spectra are illustrated in Figure 7. The general shape of the signal is in agreement with the work of Barrelle. Chemical shifts from −115 ppm to −117.3 ppm correspond to the phenol group region, whereas primary aliphatic hydroxyl groups yielded a signal between −117.3 ppm and −118.5 ppm. Moreover, in the phenol region, phenols with a C=O in the α position could be distinguished, since they led to chemical shifts between −116.2 ppm to −116.6 ppm, which is again in agreement with the observations of Barrelle.

The ^13^C and ^19^F-NMR results confirmed that the conversion of the phenolic hydroxyls of (**1**), (**2**), (**3**), and (**4**) was fully achieved, whereas that of veratryl alcohol (**5**) was poor. Therefore, the results clearly established that fluorobenzylation was efficient on phenolic hydroxyl groups, but not on aliphatic hydroxyls, and that no reaction occurred with polysaccharides.

Based on the model compounds’ reactivity towards fluorobenzylation, lignin derivatization was tested. A commercial Organosolv lignin sample (ORG) from the CIMV (Compagnie Industrielle de la Matière Végétale, France) was analyzed using ^19^F-NMR after fluorobenzylation. The ^19^F-NMR spectrum is given in Figure 7. Signal assignment was made with the help of the lignin model compounds’ ^19^F-NMR spectra. The finest signal at −118.7 ppm was assigned to FBOH, originating from the reaction of FBC with water. After ^19^F-NMR signal assignment, OH function quantification was done by a comparison of the integrals with an internal standard, i.e., 2-fluoroacetophenone, exhibiting a signal at −112.86 ppm. The integral of the sharp peak around −115.8 ppm for FBC was subtracted from the total integral value of the aromatic range. According to ^19^F-NMR quantification, the ORG lignin contained 1.71 mmol of phenolic hydroxyl groups per gram of lignin. The phenolic hydroxyl group result was in the same order as those obtained using other tested methods (see Table 6), i.e., the fast method developed in our lab (1.5 mmol/g), classical UV method (1.7 mmol/g), ^31^P-NMR (1.3 mmol/g) and conventional ^13^C-NMR after lignin acetylation (2 mmol/g). Moreover, the aminolysis method overestimated the phenolic hydroxyl group by a quantity of 2.4 mmol/g lignin, whereas ^1^H-NMR underestimated the corresponding values (0.9 mmol/g) [10]. This confirmed that ^19^F-NMR is a robust method for phenolic hydroxyl group quantification of the investigated ORG lignin.

Several NMR techniques were used for lignin hydroxyl group determination. Table 7 provides some information about the fundamental characteristics of the most common NMR techniques (^19^F, ^13^C and ^31^P-NMR). 

## 3. Materials and Methods 

### 3.1. Model Compounds

Five commercially available lignin model compounds were used: Vanillin (CAS: 121-33-5), Acetovanillone (CAS: 498-02-2), Guaiacol (CAS: 90-05-1), Vanillyl alcohol (CAS: 498-00-0) and Veratryl alcohol (CAS: 93-03-8), as well as one model of cellulose: cellobiose (CAS: 528-50-7). All were purchased from Sigma-Aldrich (Merck, Darmstadt, Germany) (Figure 2).

### 3.2. Commercial Lignin

A commercial Organosolv lignin, referred to as ORG in the present study, was purchased from the CIMV (Compagnie Industrielle de la Matière Végétale, Marne, France). The lignin was extracted from wheat straw using the CIMV process (formic acid/acetic acid/water at 185–210 °C) and the procedure can be found in the Reference [36].

### 3.3. Chemicals

Tetrabutylammonium hydroxide (1M in methanol), referred to as NBu in the present study, (CH_3_CH_2_CH_2_CH_2_)_4_N(OH) (CAS: 2052-49-5) and 4-fluorobenzyl chloride, referred to as FBC in the present study, FC_6_H_4_CH_2_Cl, (CAS: 352-11-4) were purchased from Sigma-Aldrich. Acetonitrile CH_3_CN 99.95% (CAS: 75-05-8), ethyl acetate, referred to as EtOAc in the present study, CH_3_COOC_2_H_5_ 99.5%, (CAS: 141-78-6), sodium sulfate Na_2_SO_4_, 99%, (CAS: 7757-82-6) and diethyl ether (CH_3_CH_2_)_2_O, 99.5%, (CAS: 60-29-7) were purchased from Carl Roth (Karlsruhe, Germany) and sodium chloride NaCl (CAS: 7647-14-5) and tetrahydrofuran, referred to as THF in the present study, (CH_2_)_4_O (CAS: 109-99-9) was obtained from Acros Organics (Geel, Belgium). 

### 3.4. Acetylation of the Lignin

The detailed procedure of acetylation can be found in the Reference [10].

### 3.5. Fluorobenzylation of Model Compounds

First, 100 mg of model compounds (vanillin: 0.657 mmol; acetovanillone: 0.602 mmol; guaiacol: 0.806 mmol; vanillyl alcohol: 0.649 mmol; veratryl alcohol: 0.595 mmol and cellobiose: 0.292 mmol) were dissolved in 1 mL of NBu (3.198 mmol) and stirred for 1 h at 50 °C. Then, 10 mL of acetonitrile was added, followed by 300 mg of 4-fluorobenzyl chloride (2.076 mmol) (FBC), the derivatizing agent. The reaction mixture was stirred at 50 °C for overnight. Next, distilled water (30 mL) and EtOAc (30 mL) were added to the reaction mixture. The aqueous layer was separated and extracted with EtOAc (2 × 30mL). The combined EtOAc layer was washed with distilled H_2_O (2 × 30 mL) and saturated sodium chloride solution (30 mL). The extracted EtOAc layer was dried with sodium sulfate, filtered, evaporated and analyzed without any further purification. 

### 3.6. Fluorobenzylation of the Lignin

One hundred milligrams (0.5 mmol) of lignin was used for the fluorobenzylation reaction. Lignin fluorobenzylation was performed using the same experimental conditions as for the model compounds. Derivatized lignin recovery was performed by precipitation in diethyl ether. During this process, diethyl ether might cause a loss of smaller molecular weight fragments, and especially impurities. This organic part formed a viscous precipitate, which was precipitated again in ice distilled water. This precipitate was filtered through a 0.45 µm PTFE filter, washed with distilled water several times and oven dried at 50 °C [29,30].

### 3.7. NMR

Spectroscopic measurements were recorded on a Bruker AVANCE400 spectrometer (Billerica, MA, USA) equipped with a 5 mm BB/19F-1H/d Z-GRD probe operating at 100.612 MHz for ^13^C, 376.447 MHz for ^19^F and 400.130 MHz for ^1^H. Data acquisition treatment was done using the LINUX TopSpin 3.2 software. Lignin model compound derivatives were dissolved in DMSO-d6 (40–50 mg/0.7 mL) using C_6_F_6_ as a reference (−164.90 ppm/CFCl_3_). Lignin derivatives were dissolved in DMSO-d6 (15–20 mg/0.7 mL), and quantification was done using 2-Fluoroacetophenone (3 mg) as an internal standard. The measurements were performed at 298 K. 3.75 mg of Chromium acetylacetonate was dissolved in 0.075 mL of DMSO and used as relaxing agent in 5mm NMR tubes.

### 3.8. ^19^F-NMR

The Bruker *invgate* sequence was used. The experiments were conducted with a 1.25 s acquisition time, 8.76 s relaxation delay and a 30° pulse using a 65 ppm spectral width. For data acquisition, 64k data points were used for model compounds. Prior to Fourier transformation, zero-filling at 64k was applied, followed by apodization with a 0.3 Hz exponential. Chemical shifts are given relative to CFCl_3_ (δ = 0 ppm). The positions of the peaks were referred for C_6_F_6_ as an internal reference at −164.90 ppm. For lignin OH quantification, the experiments were conducted at 298 K, with 4.35 s acquisition time, 8.76 s relaxation delay and a 30° pulse using a 20 ppm spectral width. For data acquisition, 64k data points were used. The quantification and chemical shifts of peaks were referenced with 2-fluoroacetophenone as the internal reference at −112.86 ppm.

### 3.9. ^13^C-NMR

Analyses were performed on derivatized model compounds only (the same tube was used for ^19^F and ^13^C experiments), at 298 K, using the Bruker *invgate* sequence. The experiments were conducted with a 0.648 s acquisition time, 20 s relaxation delay and a 45° pulse using a 250 ppm spectral width. Proton broad band decoupling was applied only during acquisition time. For data acquisition, 32 k data points were used. Prior to Fourier transformation, zero-filling at 64 k was applied, followed by apodization with a 2 Hz exponential. Chemical shifts are given relative to TMS (tetramethylsilane, δ = 0 ppm). The positions of the peaks were referred to the DMSO signal at 39.5 ppm.

### 3.10. ^13^C DEPT

The Bruker *dept* sequence was used. The experiments were conducted with a 0.648 s acquisition time, 3.0 s relaxation delay and a last pulse at 135° to select CH_2_ carbons reversed compared to CH and CH_3_, optimized for a 145 Hz coupling constant. (For the labelling atoms, see Figure 4).

## 4. Conclusions

This study re-investigated the ^19^F-NMR signal assignment of fluorobenzylated lignin and corrected the chemical shift values [31] using lignin-based model compounds. Fluorobenzylation reactivity was also investigated. Complete derivatization was obtained with model compounds containing only phenolic hydroxyl groups, whereas for model compounds containing both aromatic and aliphatic hydroxyls, fluorobenzylation was partial for the aliphatic group. In the case of compounds with only a primary aliphatic hydroxyl group, the derivatization was very slow and incomplete. Lignin fluorobenzylation is thus fully efficient with phenolic hydroxyl groups, but quite inefficient with aliphatic hydroxyls. To be successfully applied on lignin for aliphatic OH quantification, the derivatization conditions still have to be improved. Moreover, no fluorobenzylation was observed with a carbohydrate model compound (cellobiose), meaning that lignin sugar contamination should not interfere with the analyses. Fluorobenzylation was also applied on commercial Organosolv lignin. Based on the obtained results from the model compounds, the Organosolv lignin signal assignment and chemical shifts were aligned precisely, and the phenolic hydroxyl groups were quantified using ^19^F-NMR. The obtained phenolic hydroxyl content value was close to those acquired using other proven methods [10]. 

## Figures and Tables

**Table 1:** ^13^C-NMR chemical shifts of -CH_2_′ groups of derivatives presented in Figure 4 in different environments.

δc (in ppm)	45.3	57.54	62–63	69–71	72.81
**Methylene Group**	**-C**H_2_Cl	**-C**H_2_N	**-C**H_2_OH	**-C**H_2_OR	**-C**H_2_OMe
**Compounds**	FBC	NBu	Aliphatic OH FBOH ^(a)^	F-derivatized compounds	FBOMe ^(b)^

^(a)^ FBOH: resulting product from the reaction of FBC with water. ^(b)^ FBOMe: resulting product from the reaction of FBC with MeOH (solvent of NBu).

**Table 2:** Mixture ratio (in %) of model compound fluorobenzylation, calculated from ^13^C-NMR data (Organic part).

Compounds	Fluorobenzylation Conversion (%)	Mixture Composition (%)					
		Unreacted Starting Compound	F-Compound	FBC Reagent	FBOH	FBOMe	NBu
**1**	100	-	93.6	6.4	-	-	-
**2**	100	-	81.3	5.2	-	8.3	5.2
**3**	100	-	67.6	24.5	-	4.1	3.8
**4**	100	-	34.0	53.9	Traces	10.1	-
**5**_1 day_	9	71.5	7.1	-	Traces	-	21.4
**5**_3 days_	8	27.2	2.7	32.8	Traces	30.3	7
**6**_Organic part_	0	-	-	-	74	-	26
**6**_Aqueous part_	0	10	-	-	-	-	90
**FBC** blank		94.1	-	-	5.9	-	-
**FBC** reacted		-	-	-	7.9	-	92.1

**Table 3:** ^13^C-NMR Chemical shifts (in ppm) of fluorobenzylated model compounds.

Compounds		1	2	3	4	5
**Model Compounds Part**	**C1**	129.8	130.14	120.59	135.75	130.61
	**C2**	109.9	110.4	112.22	110.76	110.51
	**C3**	149.4	151.88	149.23	149.05	148.65
	**C4**	153	148.79	147.66	146.38	148.30
	**C5**	112.6	112.27	113.88	113.66	111.51
	**C6**	125.8	122.92	121.25	118.45	120.04
	**OCH_3_**	55.53	55.51	55.44	55.39	55.38 55.46
	**C=O**	-	196.2	-	-	-
	**HC=O**	191.34	-	-	-	-
	**CH_2_ (α)**	-	-	-	62.76	71.36
	**CH_3_**	-	26.31	-	-	-
**FBC Part**	**C′H_2_**	69.28	69.15	69.15	69.33	70.33
	**C′4**	132.5	132.74	133.44	133.54	134.71
	***^4^J_CF_*** *(Hz)*	*2.84*				
	**C′3,C′5**	130.2	130.14	129.92	129.85	129.57
	***^3^J_CF_*** *(Hz)*	*9.2*				
	**C′2,C′6**	115.3	115.29	115.15	115.15	115.40
	***^2^J_CF_*** *(Hz)*	*21.45*				
	**C′1**	161.98	161.88	161.7	161.72	161.56
	***^1^J_CF_*** *(Hz)*	*243*				

**Table 4:** Conversion and mixture ratio of model compound fluorobenzylation, calculated from ^19^F-NMR data.

Compounds	F-Compound Conversion (%)	Mixture Composition (%)			
		F-Compound	FBC Reagent	FBOH	FBOMe
**1**	100	94.5	5.5	-	-
**2**	100	86.3	6.3	-	7.4
**3**	100	72.3	23.3	-	4.4
**4**	98	38.8	55.9	1.1	10.2
**5**_1 day_	16	89.7	-	10.3	-
**5**_3 days_	12	4.9	46.0	0.6	48.5
**6**_Organic part_	0	-	-	100	-
**6**_Aqueous part_	0	-	-	-	-
**FBC**	-	99.9	-	0.1	-
**FBC** reacted	-	-	-	100	-

**Table 5:** ^19^F-NMR chemical shifts (in ppm) of fluorobenzylated model compounds.

Compounds	δ_F_ (ppm)	Nature
**1**	**−116.36**	**Φ**-OH + Aldehyde (α)
**2**	**−116.49**	**Φ**-OH + Ketone (α)
**3**	**−116.89**	**Φ**-OH
**4**	**−116.99**	**Φ**-OH
**5**	**−117.50**	**CH_2_**OH
**6**	**-**	**CH_2_**OH
**MeOH**	**−117.49**	**CH_3_**OH
**FBC (blank)**	**−115.85**	**CH_2_**Cl
**FBOH**	**−118.7**	**CH_2_**OH

**Table 6:** Comparison of phenolic hydroxyl groups (mmol/g) for Organosolv lignin using ^19^F-NMR and other techniques [10].

^19^F-NMR	Aminolysis	UV	^1^H-NMR	^13^C-NMR	^31^P-NMR	Fast Method
1.7	2.4	1.7	0.9	2.0	1.3	1.5

**Table 7:** Summary of distinctive characteristics of ^19^F, ^13^C and ^31^P-NMR.

Characteristics	^19^F	^13^C [34]	^31^P [27]
**Natural abundance**	100%	1.108%	100%
**Sample quantity**	15–20 mg	100–300 mg	30 mg
**Acquisition time**	Up to 30 min	Up to 36 h	30–120 min
**Derivatization**	Fluorobenzylation	Acetylation	Phosphitylation
**Reaction time**	1 day	1 day	in-situ reaction
**Stability of derivatized sample**	Good stability	Good stability	Not stable for a long period; requires instant acquisition
**Sugar contaminants**	No influence	Strong influence (requires high purity samples)	Strong influence (requires high purity samples)
**Structural information**	Provides structural information only for phenolic hydroxyl groups	Provides detailed structural information; Severe overlap for high molecular weight lignin	Detailed chemical information for phenolic hydroxyl groups, primary and secondary aliphatic hydroxyl groups, stereo-chemical information
**Limitations**	Poor reactive towards aliphatic hydroxyl groups	Difficult to determine side chain carbons in different lignin substructures	Expensive phosphitylating reagent (TMDP); however, it can be synthesized easily by the procedure described in Reference [35]
